# Primary extra-cranial meningioma in the right submandibular region of an 18-year-old woman: a case report

**DOI:** 10.1186/1752-1947-5-271

**Published:** 2011-07-02

**Authors:** Sanjay D Deshmukh, Vidya V Rokade, Gayatri S Pathak, Sanjana V Nemade, Amrut V Ashturkar

**Affiliations:** 1 Department of Pathology, Shrimati Kashibai Navale Medical College and General Hospital, Narhe, Pune-411041, India; 2 Department of Otolaryngology, Shrimati Kashibai Navale Medical College and General Hospital, Narhe, Pune-411041, India

## Abstract

**Introduction:**

Extra-cranial meningioma or ectopic meningioma is a rare tumor. This tumor has been reported in various anatomic sites in the head and neck, mediastinum, skin and soft tissues. We report a rare case of ectopic meningioma in the submandibular region detected by using fine-needle aspiration cytology, histopathology and immunohistochemistry. This case represents another unusual site for extra-cranial meningioma, which prompted us to report it.

**Case presentation:**

An 18-year-old Dravidian woman presented with swelling in the right submandibular region. The computed tomographic scan findings were suggestive of a neoplastic mass lesion in the right submandibular region. Fine-needle aspiration cytology led to the differential diagnosis of a monomorphic adenoma of a salivary gland or an ectopic meningioma. The patient underwent excision of the submandibular gland and tumor. The histological examination and immunohistochemistry studies confirmed that the lesion was an extra-cranial meningioma. At her two-year follow-up examination, there was no recurrence of the tumor.

**Conclusion:**

Our experience with this case indicates that, although rare, meningioma should be entertained in the differential diagnosis of a mass lesion in the head and neck region.

## Introduction

Meningiomas are among the most frequently encountered tumors of the central nervous system (CNS). They arise from arachnoid cells of the meninges. Extra-cranial primary meningioma is a tumor of rare occurrence. Ectopic meningiomas have been reported in various anatomic sites in the head and neck region, such as the floor of the mouth [1], the nose and the paranasal sinuses [2]. In addition, ectopic meningiomas have been reported in other rarer sites such as the lung, mediastinum, skin, retroperitoneum and thigh [3]. At these sites, they are believed to arise from the arachnoid cells along the peripheral nerves [3]. Though ectopic meningiomas at these rare sites may pose diagnostic difficulties for clinicians and cytologists, the diagnosis of this condition is of paramount importance, as surgical excision is curative.

## Case presentation

**Figure 1:**
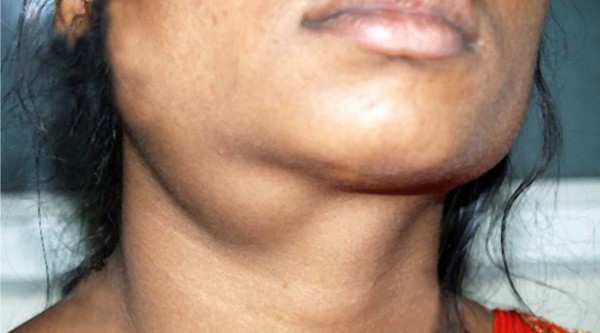
**Clinical photograph showing a mass in the right submandibular region**.

**Figure 2:**
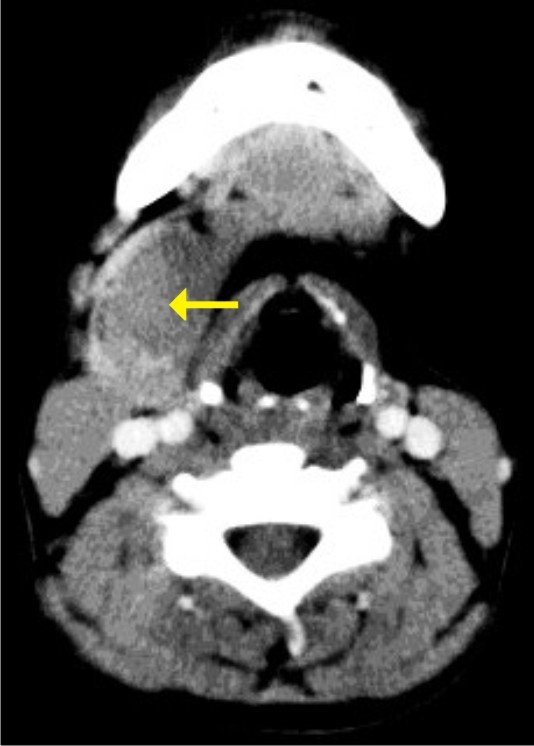
**Computed tomographic scan showing a minimally enhancing hypodense lesion (arrow) within the right submandibular salivary gland with an enhancing rim of the normal submandibular gland at the periphery**.

**Figure 3:**
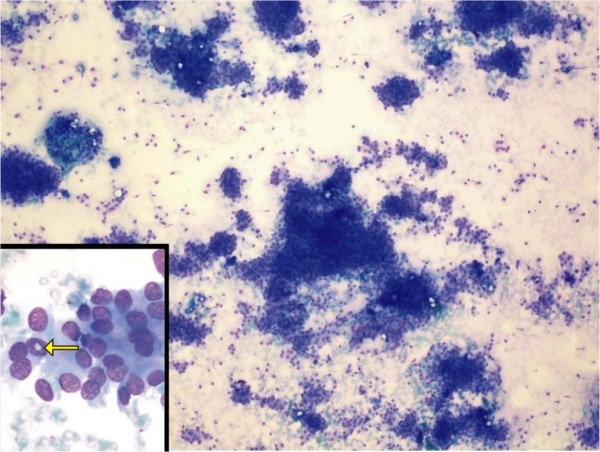
**Photomicrograph showing moderately cellular smears composed of cells arranged in loose clusters, sheets and occasional whorls (hematoxylin and eosin stain; original magnification, ×100)**. Inset shows intra-nuclear inclusion (arrow).

**Figure 4:**
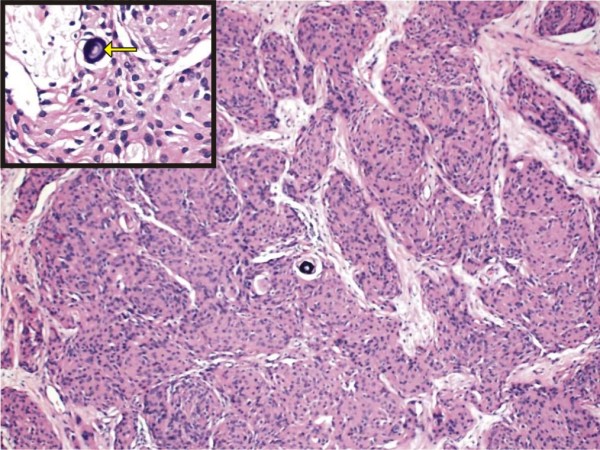
**Photomicrograph showing cells arranged in short fascicles and concentric whorls and at places in a typical meningothelial pattern (hematoxylin and eosin stain; original magnification, ×100)**. Inset shows a closer view of the psammoma body (arrow).

**Figure 5:**
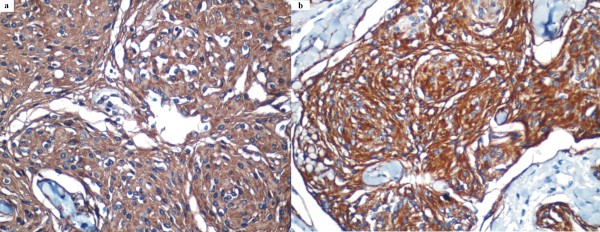
**Cells showing strong cytoplasmic immunostaining for **(a) **epithelial membrane antigen and **(b) **vimentin (original magnification, ×400)**.

An 18-year-old Dravidian woman presented to our hospital with right submandibular swelling of three to four months' duration. An examination revealed a firm, non-tender swelling in the right submandibular region measuring 3 cm × 3 cm (Figure 1). Computed tomographic (CT) scan findings were a minimally enhancing, hypodense lesion measuring 4.5 cm × 3.7 cm × 2.6 cm within the right submandibular salivary gland, with an enhancing rim of the normal submandibular gland at the periphery (Figure 2). The CT scan revealed no evidence of the mass lesion elsewhere in the head and neck region. Fine-needle aspiration was done, which showed moderately cellular smears composed of cells arranged in loose clusters and sheets showing a whorling pattern in places. Individual cells were polygonal to spindle-shaped with abundant eosinophilic cytoplasm. The nuclei were round to ovoid and regular with finely granular, evenly distributed chromatin. Few cells showed intra-nuclear cytoplasmic inclusion (Figure 3). On the basis of these findings, a diagnosis of right submandibular gland neoplastic lesion, suggestive of oncocytoma, was made. The possibility of a primary ectopic meningioma was also considered on the basis of the findings of the whorling arrangement of cells and intra-nuclear inclusion. Intra-operatively, the submandibular salivary gland could be dissected separately, and the well-circumscribed tumor was seen in the vicinity of the deep lobe of the gland, which was surgically excised. Grossly, two tissue masses were removed. The larger mass was smooth, firm, well circumscribed and oblong, measuring 3 cm × 3 cm × 2 cm. The cut surface was grayish white with few areas of congestion. The smaller mass was a salivary gland. The cut surface of the gland appeared unremarkable. Multiple sections were taken from the tumor. Histologically, the lesion was characterized by a lobular architecture and showed uniform spindle-cell proliferation separated by hyalinized collagen bundles. The cells were arranged in short fascicles, in concentric whorls and, in places, in a typical meningothelial pattern (Figure 4). The neoplastic cells had abundant, lightly eosinophilic cytoplasm, indistinct cytoplasmic borders and round or oval nuclei with finely dispersed chromatin and indistinct nucleoli. A careful search revealed psammoma bodies. Immunohistochemistry (IHC) was performed using the following pre-diluted, ready-to-use primary antibodies: epithelial membrane antigen (EMA) (clone E29; Dako, Carpinteria, CA, USA), vimentin (clone V-9; BioGenex, Hyderabad, India cytokeratin (CK) (clone AE-1/AE-3; Dako) and S-100 protein (polyclonal antibody S100A4; Dako). The tumor cells showed intense reactivity for EMA and vimentin, but not for CK or S-100 protein (Figure 5). On the basis of these findings, a diagnosis of primary extra-cranial ectopic meningioma was made.

## Discussion

Ectopic meningiomas represent a well-known entity and are reported to occur in different locations. Meningiomas are common tumors of the CNS that arise from the arachnoid cells of the meninges. Rarely, these meningiomas appear in extra-cranial and extra-spinal areas, where they are known as ectopic meningiomas. Their reported incidence ranges from 0.9% to 2.0% of all meningiomas [4]. Heterotopic brain and meningeal tissues have been known to occur occasionally in the mid-line of head, neck and trunk as a result of the displacement of such tissue during the fusion of skull and spine in the embryonic state [5]. In two of three cases, neck localization is a result of connection to a cranial or spinal meningioma [6]. Four mechanisms of the formation of ectopic meningioma have been suggested: (1) direct extension of an intra-cranial lesion, (2) distant metastasis from an intra-cranial meningioma, (3) origination from arachnoid cells within the sheaths of cranial nerves and (4) origination from embryonic nests of arachnoid cells [7].

However, primary ectopic meningiomas are very rare and have been reported in the orbit [5], head and neck region, lung, mediastinum, skin, retroperitoneum, thigh muscle and foot [3]. A primary ectopic meningioma of the external auditory canal was also reported [8].

In our patient, the clinical impression was that of a salivary gland neoplasm because of its location in the submandibular area. On the basis of fine-needle aspiration cytology (FNAC), the differential diagnosis of oncocytoma and extra-cranial meningioma was made. The cells were oval to elongated and were arranged in loose clusters with a whorled appearance in places. Intra-nuclear pseudo-inclusions were also observed. Similar experiences at the time of FNAC-based diagnosis have been reported by others [1]. Under light microscopy, the cells were arranged in a meningothelial pattern, which is known to be common in ectopic meningiomas [4,5]. The findings of psammoma bodies in our patient further strengthened the diagnosis. Four microscopic patterns of meningiomas are recognized: (1) the syncytial type, consisting of a uniform sheet of polygonal cells; (2) a transitional or psammomatous form with a whorled pattern of spindle cells and psammoma bodies; (3) a fibrous form with reduced cellularity and increased collagen content; and (4) the angioblastic type with high cellularity and an adjacent syncytial or transitional form. Most extra-cranial meningiomas are of the syncytial or transitional form [9].

IHC analyses of the reported cases of primary ectopic meningioma are similar to their intra-cranial counterparts [10]. The tumor cells showed intense reactivity for EMA and vimentin, but not for CK and S-100 protein, findings that are consistent with the diagnosis of meningioma [11]. Taking into account the cytological features and the light microscopy and IHC findings in our patient, a diagnosis of ectopic meningioma was made.

The treatment of choice for extra-cranial meningiomas is surgical excision. The patient's prognosis is good after complete resection. All of the previously reported cases have been treated in this way, and given the long evolution and the lack of recurrence in all of them, it may be concluded that this lesion behaves in a non-aggressive fashion [12].

## Conclusion

To summarize, ectopic meningiomas can pose a diagnostic difficulty for the clinician as well as for the cytopathologist, as it is a diagnosis which may be easily forgotten in the list of differential diagnoses of neck masses. Although these tumors are unusual, their characteristic histological features establish the diagnosis.

## Consent

Written informed consent was obtained from the patient for publication of this case report and any accompanying images. A copy of the written consent is available for review by the Editor-in-Chief of this journal.

## Competing interests

The authors declare that they have no competing interests.

## Authors' contributions

SD had a major role in establishing the diagnosis histologically and in critically evaluating and revising the manuscript. GP performed the histopathological examination of the specimen and was involved in drafting the manuscript. VR treated the patient surgically and read and approved the revised manuscript. SN managed the patient and provided the data. AV participated in the literature review and contributed to the compiling and editing of the data. All authors have read and approved the final manuscript.
